# MicroRNA-351 Regulates Two-Types of Cell Death, Necrosis and Apoptosis, Induced by 5-fluoro-2'-deoxyuridine

**DOI:** 10.1371/journal.pone.0153130

**Published:** 2016-04-12

**Authors:** Akira Sato, Takuya Omi, Akihiro Yamamoto, Akito Satake, Akiko Hiramoto, Mitsuko Masutani, Sei-ichi Tanuma, Yusuke Wataya, Hye-Sook Kim

**Affiliations:** 1 Department of Biochemistry, Faculty of Pharmaceutical Sciences, Tokyo University of Science, Yamazaki, Noda, Chiba, Japan; 2 Division of Genome Stability Research, National Cancer Center Research Institute, Tsukiji, Chuo-ku, Tokyo, Japan; 3 Division of Chemotherapy and Clinical Research, National Cancer Center Research Institute, Tsukiji, Chuo-ku, Tokyo, Japan; 4 Department of Drug Informatics, Faculty of Pharmaceutical Sciences, Okayama University, Tsushima-naka, Kita-ku, Okayama, Japan; 5 Department of Frontier Life Sciences, Nagasaki University Graduate School of Biomedical Sciences, Sakamoto, Nagasaki, Japan; University of Saarland Medical School, GERMANY

## Abstract

Cell-death can be necrosis and apoptosis. We are investigating the mechanisms regulating the cell death that occurs on treatment of mouse cancer cell-line FM3A with antitumor 5-fluoro-2'-deoxyuridine (FUdR): necrosis occurs for the original clone F28-7, and apoptosis for its variant F28-7-A. Here we report that a microRNA (miR-351) regulates the cell death pattern. The miR-351 is expressed strongly in F28-7-A but only weakly in F28-7. Induction of a higher expression of miR-351 in F28-7 by transfecting an miRNA mimic into F28-7 resulted in a change of the death mode; necrosis to apoptosis. Furthermore, transfection of an miR-351 inhibitor into F28-7-A resulted in the morphology change, apoptosis to necrosis, in this death-by-FUdR. Possible mechanism involving lamin B1 in this miR-351’s regulatory action is discussed.

## Introduction

MicroRNAs (miRNAs) are endogenous small non-coding RNAs, 21–25 nucleotides-long, that function as gene silencers by binding to the 3’-untranslated region (UTR) of target mRNAs, inhibiting initiation of protein synthesis and/or promoting mRNA cleavage [1–3]. In addition, recent reports indicate that several miRNAs interact with its target mRNA in 5’-UTR or coding sequence (CDS), regulating post-transcription [4–7]. MiRNAs regulate many biological processes including cell development, differentiation, and cell death [3, 8, 9]. MicroRNA-351 (miR-351) is one of the interferon beta (INFβ)-inducible mRNAs, and is known to promote cellular antiviral activities [10]. In addition, miR-351 regulates development of the nerve-system [11] and promotes muscle regeneration [12] by targeting transmembrane proteins 59 (TMEM59) and E2f3, respectively.

For cell death, two major processes have been characterized, i.e., necrosis and apoptosis, according to morphological features [13–15]. We have investigated the molecular mechanisms regulating the necrosis in original F28-7 and apoptosis in its subclone variant F28-7-A that occur on treatment of mouse mammary carcinoma FM3A cells with 5-fluoro-2’-deoxyuridine (FUdR) [16–23]. These two-types of cell death, necrosis in F28-7 and apoptosis in F28-7-A, after treatment with FUdR were recognizable by observing cell death morphology. Necrosis in F28-7 is characterized by the swelling of the cell and organelles, and by the disruption of cellular and nuclear membranes [18]. In addition, necrosis is accompanied by cleavage of the apoptosis marker proteins caspase-3 and poly(ADP-ribose) polymerase-1 (PARP-1), and breakdown of DNA into chromosome-sized fragments [18, 23]. In contrast, apoptosis in F28-7-A is characterized by membrane blebbing, shrinking of the cell and its organelles, the cleavage of caspase-3 and PARP-1, and oligonucleosomal degradation of DNA [18, 23]. Previously, we reported three possible regulators in the processes of cell death; necrosis and apoptosis: *i*.*e*., the nuclear scaffold lamin-B1, the cytoplasmic intermediate filament cytokeratin-19, and the transcription factor ATF3. These cell-death regulators were discovered by proteomic and transcriptomic analyses of dying cells with the use of the small interfering-RNA approach [20–23]. In the present study, to understand the molecular mechanisms underlying the two types of cell death, necrosis and apoptosis, we investigated the miRNA expression profiles of necrosis-fated F28-7 and apoptosis-fated F28-7-A cells.

Here, we show that the miR-351 can participate in regulating cell death morphology. We found that a higher expression of miR-351 in the FUdR-treated death-destined cell caused a shift from the necrosis to apoptosis. Conversely, transfection of an miR-351 inhibitor into F28-7-A resulted in a switch from apoptosis to necrosis.

## Materials and Methods

### Reagents, and cell culture

5-Fluoro-2′-deoxyuridine (FUdR) was obtained from Sigma. FUdR was stored as 2 mM stocks in ultra pure water at −20°C. Original-type F28-7 clone and variant F28-7-A clone of mouse mammary carcinoma FM3A cells used in the study were those described previously [18]. The FM3A cells were maintained by suspension culture. These cells were grown at 37°C under a humidified 5% CO_2_ atmosphere in ES medium containing 2% heat inactivated fetal bovine serum. F28-7 and F28-7-A cells (approximately 5×10^5^ cells/ml) were treated with 1 μM FUdR. Cell viability was estimated with a hemocytometer by means of trypan blue-exclusion.

The miRNA-351 mimic was Syn-mmu-miR-351-5p miScript miRNA mimic (Catalog number: MYS0000609, QIAGEN), and the nonsilencing siRNA was AllStars Negative Control siRNA (Catalog number: 1027280, QIAGEN). The miRNA-351 inhibitor was miScript miRNA inhibitor Anti-mmu-miR-351-5p (Catalog number: MIN0000609, QIAGEN), and the negative control miRNA inhibitor was miScript Inhibitor Negative Control (Catalog number: 1027271, QIAGEN). The primers used were, Mm_miR-351_1 miScript Primer Assay (Catalog number: MS00002219), Mm_miR-700_1 miScript Primer Assay (MS00002898), Mm_miR-743a_1 miScript Primer Assay (MS00012649), Mm_miR-140*_1 miScript Primer Assay (MS00006048), Mm_miR-222_2 miScript Primer Assay (MS00007959), Mm_miR-34c*_1 miScript Primer Assay (MS00011907), Mm_miR-132_1 miScript Primer Assay (MS00001561), and Hs_RNU6-2_11 miScript Primer Assay (MS00033740), purchased from QIAGEN. The primary antibodies; mouse monoclonal anti-lamin-B1, anti-human cytokeratin-19, anti-ATF3 (C-19), anti-HMGB1, and rabbit polyclonal anti-glyceraldehyde-3-phosphate dehydrogenase (GAPDH) antibodies were from Zymed Laboratories, ANASPEC, Santa Cruz Biotechnology, Abcam, and Trevigen, respectively. The secondary antibodies; anti-mouse IgG horseradish peroxidase-linked whole antibody and anti-rabbit IgG horseradish peroxidase-linked whole antibod were from GE Healthcare. 4,6-Diamidino-2-phenylindole dihydrochloride (DAPI) was purchased from Invitrogen.

### RNA extraction and microarray analysis

For microarray analysis, the total RNA fraction was isolated from the individual cell lines using the QIAshredder spin columns and an RNeasy Mini kit, as described by the manufacturer (QIAGEN). Biotin-labeled RNA was prepared using a Flash Tag^TM^ Biotin RNA Labeling Kit according to the manufacturer’s instructions (Genisphere). The labeled RNA product was mixed in hybridization cocktail. The hybridization cocktails were added to GeneChip^®^ miRNA 2.0 Array (Affymetrix) in the GeneChip Hybridization Oven 640 under constant rotation at 60 rpm at 48°C for 16 h. After the hybridization, GeneChips were washed and stained with GeneChip Fluidics Station 450 (Affymetrix), and scanned with a GeneChip Scanner 3000 7G (Affymetrix). Scanned GeneChip images were analyzed using GeneChip Command Console software (AGCC) and miRNA QCTool (Affymetrix). Microarray data were analyzed using GeneSpring software (Agilent technologies).

### Quantitative real-time PCR for mature miRNAs

Total small RNA fraction was extracted with an miRNeasy mini kit (QIAGEN) according to manufacturer’s instructions. Then, cDNA samples were reverse-transcribed using 1 μg total small RNA, miScript II RT kit (QIAGEN) according to instructions. The expression levels of mature miRNA were analyzed using miScript SYBR Green PCR kit (QIAGEN) and LightCycler system (Roche). Mature miRNA levels were normalized to RNU6B and quantified using the comparative *Ct* method.

### Transfection for miRNA mimic and inhibitor

Exponentially growing 2×10^5^ cells were suspended in 75 μl siPORT electroporation buffer (Ambion) containing miR-351 mimic, non-silencing siRNA, miR-351 inhibitor or negative control miRNA inhibitor (final concentration 8×10^−7^ M) and introduced into a 0.1-cm gap electroporation cuvette (Bio-Rad). Cells were then electroporated using the Bio-Rad Gene Pulser Xcell at voltage 0.15 kV, pulse length 1,000 μs, and number of pulse 1. After electroporation, cells were plated at 5×10^4^ cells/ml in fresh ES medium in tissue culture flasks. Forty-eight hours after the electroporation, cells were used for further experiments. Transfection efficiencies at higher than 80% were obtained in this electroporation protocol by using the nonsilencing siRNA conjugated with Alexa Fluoro 488 [see ref. [20] for the conditioning].

### Western blotting

Western blot analysis was performed as described previously [20, 22, 23]. The following antibodies were used: anti-ATF3 antibody (1:200), anti-lamin-B1 antibody (1:1,500), anti-cytokeratin-19 antibody (1:200), anti-HMGB1 antibody (1:1,000), anti-GAPDH antibody (1:10,000), anti-mouse IgG horseradish peroxidase-linked whole antibody (1:20,000), and anti-rabbit IgG horseradish peroxidase-linked whole antibody (1:20,000).

### Morphological observation

The morphological observation experiments, namely, cell fixation and staining were performed as described previously [22]. Cell morphology was observed by Olympus BX61 and Leica microsystems DMI6000B fluorescence microscopes.

### Statistical analysis

Determination of the significance of differences among groups was done using the Student’s *t*-test. *P*<0.05 was considered significant.

## Results and Discussion

### MicroRNA expression profiles of the necrosis-fated F28-7 and the apoptosis-fated F28-7 cells

**Fig 1:**
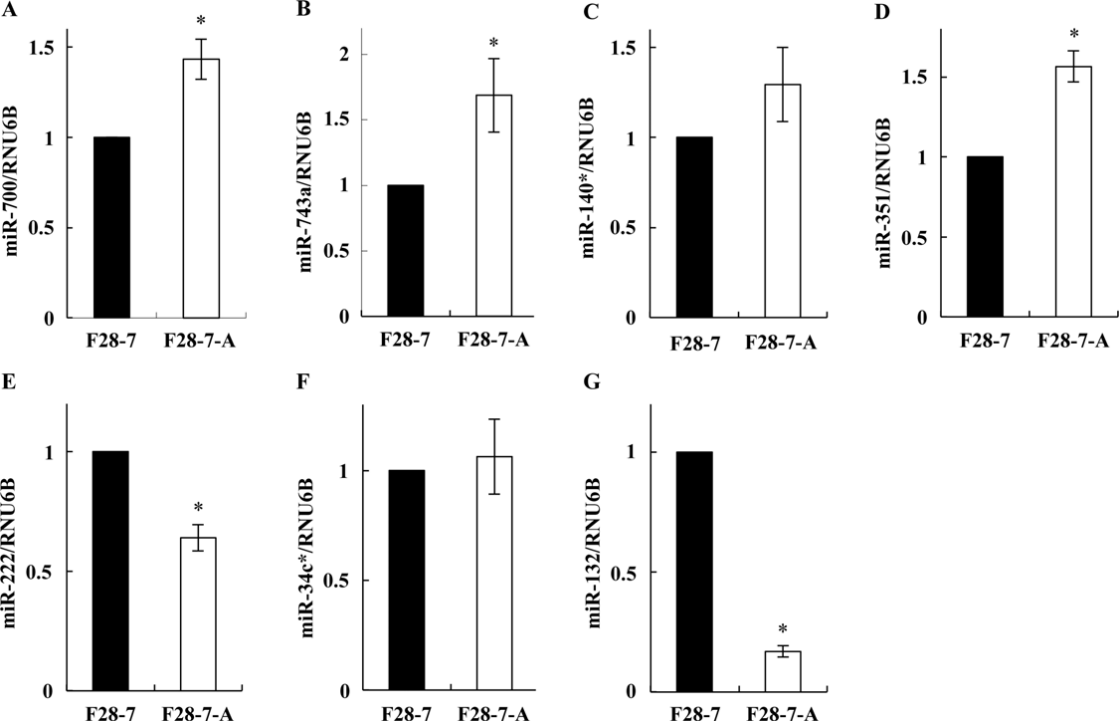
Quantitative real-time PCR validation of differentially expressed mature miRNAs. Total small RNA fractions were prepared from F28-7 and F28-7-A cells (no drug, no incubation). Expression of (A) miR-700, (B) miR-743a, (C) miR-140*, (D) miR-351, (E) miR-222, (F) miR-34c*, (G) miR-132, and RNU6B were analyzed by quantitative real-time PCR using primers for miR-700-3p, miR-743a-3p, miR-140-3p, miR-351-5p, miR-222-3p, miR-34c-3p, miR-132-3p, and RNU6B (see Materials and Methods). The expression of RNU6B was used as an internal control. Results are averages of three independent experiments with error bars showing the ±SD in triplicates. The asterisk indicates a statistically significant difference (Student’s *t* test, *p<0.05, **p<0.01).

First, we investigated the miRNA expression profiles in these sister cells at the untreated stage (no drug), by microarray analyses. For both F28-7 and F28-7-A cells at the untreated stage, three independent analyses were performed with the Affymetrix GeneChip miRNA 2.0 Array, which contains 722 probes set for mouse mature miRNAs and 690 probes set for mouse pre-miRNA, allowing analyses of the expression levels of mouse mature miRNAs. Using 1.5-fold cut-off, the analysis identified seven differentially expressed miRNAs: in F28-7-A (apoptosis-fated cell), four mature miRNAs (miR-700, miR-743a, miR-140*, miR-351) were expressed at higher levels, and three mature miRNAs (miR-222, miR-34c*, miR-132) were expressed at lower levels than in F28-7 (necrosis-fated cell). Table 1 lists the names and database information of these seven mature miRNAs. Next, to verify the microarray studies, we analyzed the expression levels of these mature miRNAs by quantitative real-time PCR. As shown in Fig 1A–1D, miR-700, miR-743a, miR-140*, and miR-351 were expressed at higher levels in F28-7-A than in F28-7 cells. In addition, miR-222 and miR-132 were expressed at lower levels in F28-7-A than in F28-7 cells (Fig 1E and 1G). It was noted that the expression levels of miR-34c* were similar in these sister cells (Fig 1F). Therefore, we assumed that the miR-700, miR-743a, miR-140*, miR-351, miR-222, and miR-132 were candidate miRNAs as cell-death regulators in necrosis and apoptosis.

**Table 1:** Differentially expressed miRNAs in F28-7 and F28-7-A cells. **Note:** *a* miRBase primary accession number. *b* Fold difference (F28-7-A/F28-7 cells) > 1.5 or ≤ 0.6 (p < 0.05).

**Accession number** ^***a***^	**ID**	**MicroRNA name**	**Sequence**	**Fold difference** ^***b***^
MIMAT0003490	mmu-miR-700-3p	**mmu-miR-700**	CACGCGGGAACCGAGUCCACC	1.6
MIMAT0004238	mmu-miR-743a-3p	**mmu-miR-743a**	GAAAGACACCAAGCUGAGUAGA	1.6
MIMAT0000152	mmu-miR-140-3p	**mmu-miR-140-star**	UACCACAGGGUAGAACCACGG	1.7
MIMAT0000609	mmu-miR-351-5p	**mmu-miR-351**	UCCCUGAGGAGCCCUUUGAGCCUG	1.8
**Decreased in F28-7-A**				
**Accession number** ^***a***^	**ID**	**MicroRNA name**	**Sequence**	**Fold difference** ^***b***^
MIMAT0000670	mmu-miR-222-3p	**mmu-miR-222**	AGCUACAUCUGGCUACUGGGU	0.6
MIMAT0004580	mmu-miR-34c-3p	**mmu-miR-34c-star**	AAUCACUAACCACACAGCCAGG	0.6
MIMAT0000144	mmu-miR-132-3p	**mmu-miR-132**	UAACAGUCUACAGCCAUGGUCG	0.5

### Modulation of FUdR-induced necrosis and apoptosis, by miR-351

**Fig 2:**
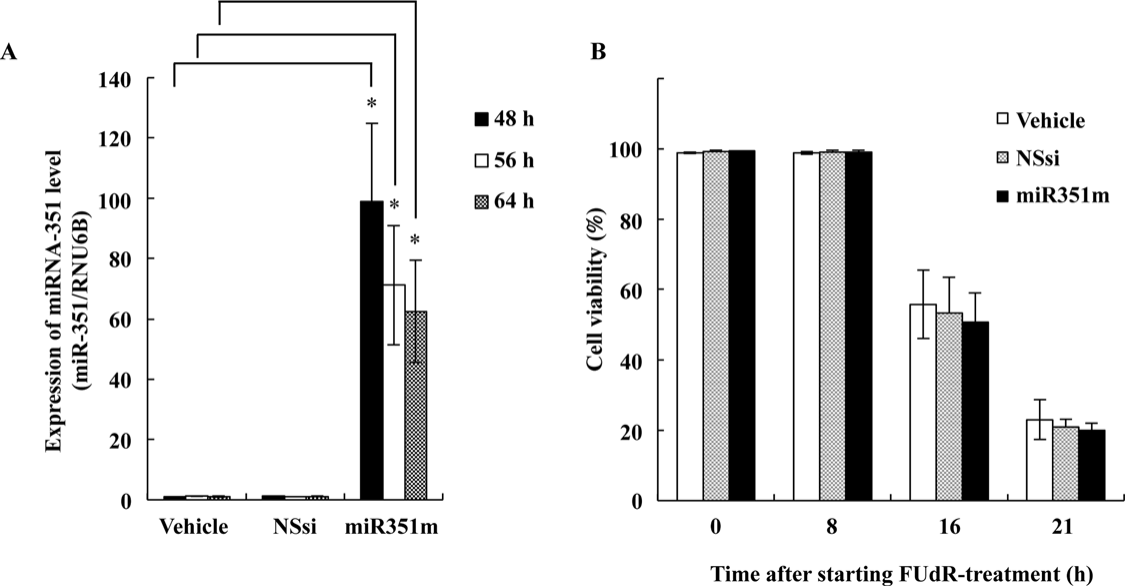
Higher expression of miR-351 by synthetic miR-351 mimic-transfection. (A) F28-7 cells were transfected with nonsilencing siRNA (NSsi), and with mature miR-351-5p mimic (miR351m). At 48, 56, and 64 hours after the transfection, the levels of miR-351-5p and RNU6B (an internal standard) were analyzed by quantitative real-time PCR. Results are averages of three independent experiments with error bars showing the ±SD in triplicates. The asterisk indicates a statistically significant difference (Student’s *t* test, *p<0.005). (B) Forty-eight hours after transfection, F28-7 cells were treated with 1 μM FUdR for 0, 8, 16, and 21h. The cell viability was examined by trypan-blue exclusion. Values are mean ± SD for three independent experiments.

**Fig 3:**
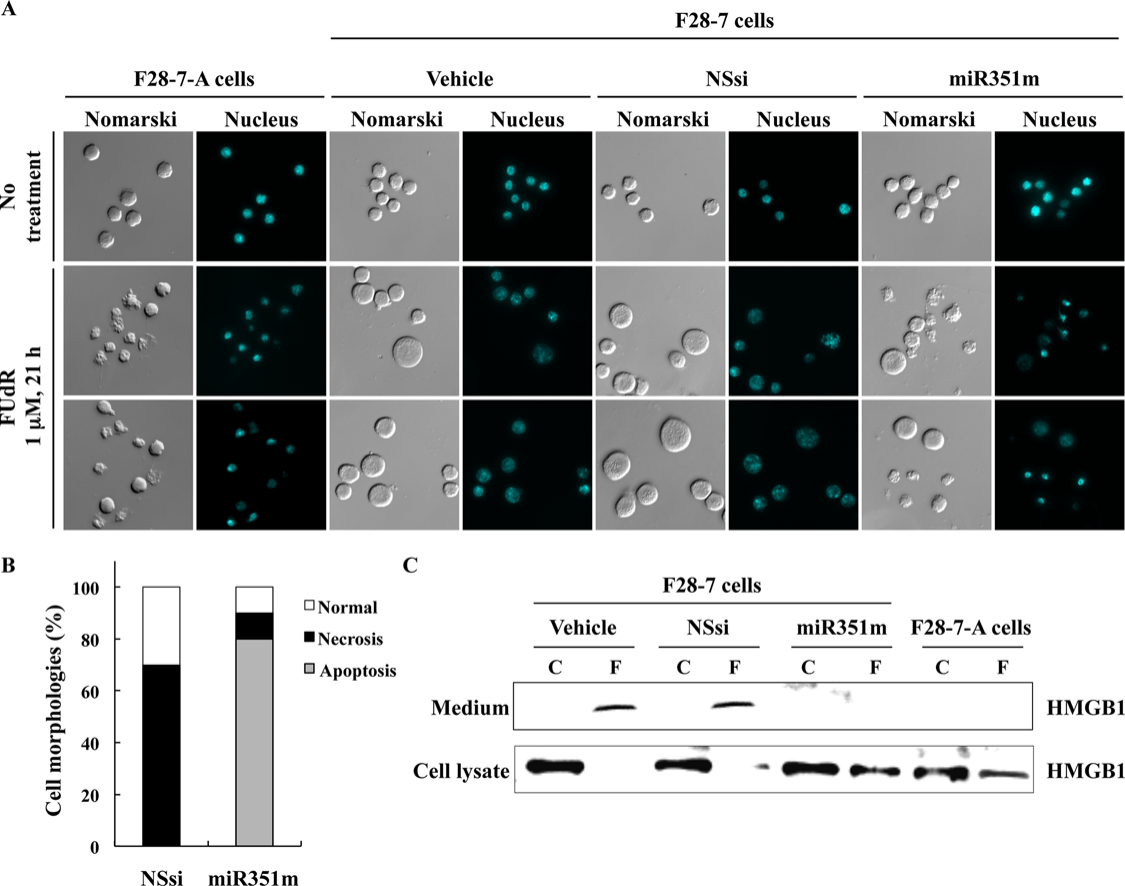
Higher expression of miR-351 shifts the FUdR-induced cell death morphologies from necrosis to apoptosis. (A) At 48 hours after transfection with the vehicle, non-silencing siRNA (NSsi) or the mature miR-351-5p mimic (miR351m), the F28-7 cells were treated with or without 1 μM FUdR for 21 h, and then stained with DAPI. Morphological changes were analyzed by Olympus BX61 fluorescence microscope at 400× magnification. (B) Percentage of necrosis, apoptosis, and normal cell morphologies in FUdR-treated F28-7 cells transfected with NSsi or miR351m. (C) At 48 hours after transfection with the vehicle, non-silencing siRNA (NSsi) or the miR-351 mimic (miR351m), the F28-7 cells were treated with or without 1 μM FUdR for 21 h, and analyzed by western blotting for anti-HMGB1 antibody. As a negative control, F28-7-A cells were treated with or without 1 μM FUdR for 21 h, and examined by western blotting. Two additional independent experiments gave similar results.

To test whether inhibition/overexpression of these candidate miRNAs in the F28-7 cells modulate FUdR-induced cell death, we have done transfections of the miRNA inhibitors and/or the synthetic miRNA mimics. We found that the higher expression of miR-351 in F28-7 by the synthetic miR-351 mimic caused a shift from necrosis to apoptosis. Quantitative real-time PCR analyses performed for total small RNA fraction in F28-7 at 48, 56, and 64 h after the transfection indicated that the synthetic miRNA mimic-treatment resulted in a higher expression of the miRNA-351 (Fig 2A). The expression efficacy of miR-351 became more than 100-fold at 48 h in miR-351 mimic-transfected F28-7 cells, compared to that in the non-silencing siRNA-transfected cells. The expression level of miR-351 in F28-7 cells significantly decreased in a time-dependent manner. It may be that the overexpressed miR-351 could have received exclusion or degradation in the F28-7 cells. As shown in Fig 2B, the transfection of miR-351 mimic into the F28-7 cells did not change the cell viability. In addition, either the non-silencing siRNA alone or the miR-351 mimic alone had no effect on the cell morphology; *i*.*e*., the morphology-change required subsequent FUdR-treatment (Fig 3A, upper diagram).

We explored the morphology of the F28-7 cells possessing high miR-351 expression on treatment with FUdR. The necrotic morphology in F28-7 and apoptotic morphology in F28-7-A cells were characteristically observed at 21 h after the treatment with FUdR. At the 21 h, the controls given vehicle or non-silencing siRNA showed the swelling, a feature of necrosis. In contrast, the synthetic miR-351 mimic-transfected cells showed a typical apoptotic morphology; the chromatin condensation and the cell bleb (Fig 3A, at the bottom). It was noted that the nucleus of FUdR-induced necrotic morphologies was slightly stained with the fluorescent DNA dye DAPI. In addition, microscopic observation showed that the distribution of cell morphologies was 10% normal, 10% necrosis, and 80% apoptosis in the miR-351 transfected F28-7 cells. These data were obtained by counting more than 300 cells in the microscopic examination. Importantly, transfection efficiencies higher than 80% were obtained by the electroporation protocol using the nonsilencing-siRNA conjugated with Alexa Fluoro 488 [20]. In our microarray analyses, RNA metabolism-related genes (for example, 2-5A-dependent ribonuclease RNase L) were found to have increased in the FUdR-induced necrosis [21]. The 10% necrosis in this experiment might have been caused by unsuccessful transfection of the miR-351 mimic, or by degradation of the transfected miRNA. On the other hand, the almost all of the dying cells underwent necrotic cell morphologies after vehicle or non-silencing siRNA transfection with subsequent FUdR-treatment. Our results demonstrated that the high miR-351 expression shifts FUdR-induced necrotic morphologies into apoptotic morphologies. Because clonogenic ability of FM3A cells was low, we examined cell growth and viability by trypan-blue dye exclusion. At this point of treatment, the cell viability was 23% (n = 3) with the vehicle, 21% (n = 3) with the non-silencing siRNA, and 20% (n = 3) with the miR-351 mimic transfection (Fig 2B). We confirmed, by western blot analysis for high mobility group box 1 (HMGB1) in culture medium, a known indicator of necrotic cell death [24, 25], that the leakage of HMGB1 from nucleus to culture medium in FUdR-induced necrosis became almost null by the transfection of miR-351 mimic (Fig 3B). These observations indicate that the expression of miR-351 plays a key role in FUdR-induced apoptosis.

**Fig 4:**
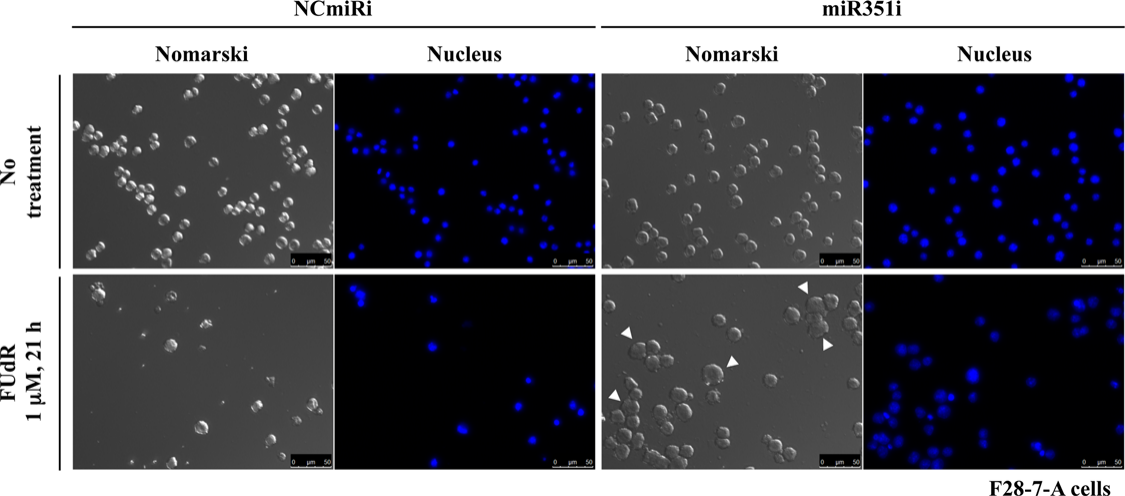
Transfection of miR-351 inhibitor in F28-7-A shifts the FUdR-induced cell-death morphology from apoptosis to necrosis. Forty-eight hours after transfection with the negative control miRNA inhibitor (NCmiRi) or the miR-351 inhibitor (miR351i), the F28-7-A cells were treated with or without 1 μM FUdR for 21 h, and then stained with DAPI as described in Materials and Methods. Morphological changes were analyzed by Leica DMI6000B-AFC fluorescence microscope at 400× magnification. The arrowhead indicates necrosis cells. Scale bar = 50 μm.

Next, we analyzed the inhibition of miR-351-5p in F28-7-A cells using miR-351-5p targeted miRNA inhibitor. As shown in Fig 4, the miR-351 inhibitor-transfected cells underwent a typical necrotic morphology (cell swelling; indicated by the arrowhead) at 21 h after treatment with FUdR. On the other hand, F28-7-A cells transfected with negative control miRNA inhibitor showed membrane blebbing and chromatin condensation, typical of apoptosis. Thus, a transfection of miR-351 inhibitor shifted the FUdR-induced apoptotic morphology into necrotic morphology in the apoptosis-fated F28-7-A cells.

Furthermore, this phenotypic screening by using miRNA mimics showed that the higher expression of miR-743a in F28-7 by the synthetic miR-743a mimic can cause a shift from FUdR-induced necrosis to apoptosis (S1 Fig). These findings suggest that the expression of miR-743a plays a key role in FUdR-induced apoptosis. It is important to further investigate the relationship between the miR-743a expression and these two-types of cell death, necrosis and apoptosis.

### Association of miR-351 and the nucleus-scaffold protein lamin-B1 expression

**Fig 5:**
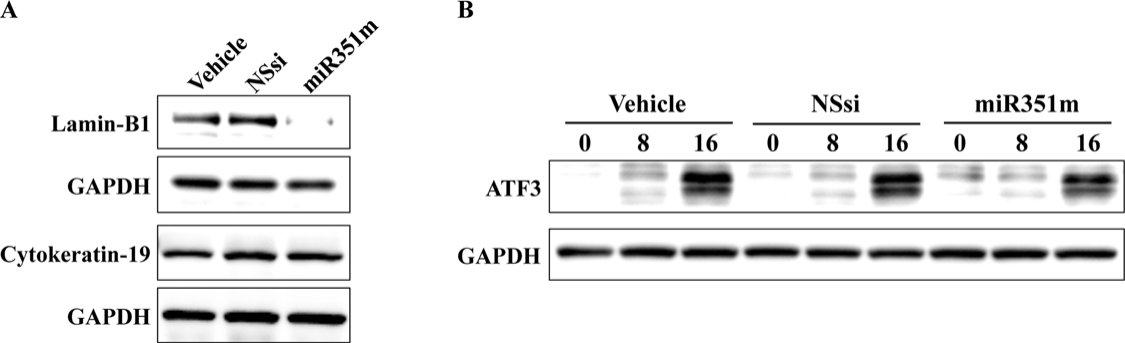
Higher expression of miR-351 modulates the expression of the nucleus intermediate filament lamin-B1. (A) At 48 hours after transfection with the vehicle, non-silencing siRNA (NSsi) or the mature miR-351-5p mimic (miR351m), the F28-7 cells were examined by western blotting for anti-lamin-B1, cytokeratin-19, and GAPDH. (B) At 48 hours after transfection with the vehicle, non-silencing siRNA (NSsi) or the mature miR-351-5p mimic (miR351m), the F28-7 cells were treated with 1 μM FUdR for 0 (no drug), 8, 16 h, and then analyzed by western blotting for anti-ATF3 and GAPDH antibody. The expression of GAPDH was used as an internal control. Two additional independent experiments gave similar results.

We previously identified three new regulators of cell death: nuclear scaffold protein lamin-B1 [20], cytoplasmic scaffold protein cytokeratin-19 [22], and transcription factor ATF3 [23], by using proteomic, transcriptomic analyses and siRNA screening. To test if an higher expression of miR-351 in F28-7 can modulate the expression levels of these three regulators, we investigated the protein levels of lamin-B1, cytokeratin-19, and ATF3 using western blot analysis. As shown in Fig 5A, the transfection of miR-351 mimic in F28-7 led to reduction of lamin-B1 protein without any reduction of cytokeratin-19. In addition, FUdR-induced ATF3 induction was not affected by the transfection of miR-351 mimic (Fig 5B). The GAPDH protein levels as a control showed no change by the transfection of miR-351 mimic (Fig 5A and 5B). These findings suggest that miR-351 could regulate nuclear scaffold lamin-B1 expression and mediate FUdR-induced apoptosis.

**Fig 6:**
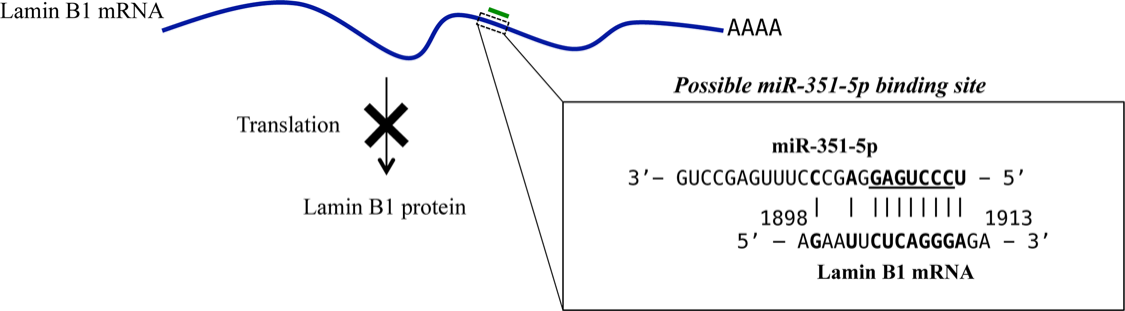
Possible association site for miR-351-5p and lamin-B1 mRNA. By examining the available sequence information (miR-351-5p, accession number MIMAT0000609 in miRBase; lamin-B1 mRNA, accession number NM_010721 in NCBI), we became aware that these two RNAs may interact in the coding region of lamin-B1 mRNA, resulting in the inhibition of protein synthesis. Complementary pairs of up to ten nucleotides are present between the messenger and the microRNAs. Such a coding-region association may give rise to translation blockage [1, 2].

Next, we analyzed the protein levels of lamin-B1 in FUdR-induced necrosis and apoptosis by western blotting. The level of lamin-B1 protein was indeed higher in F28-7 than in F28-7-A in untreated stage (S2 Fig). In the F28-7 cells, the level of lamin-B1 protein slightly increased until 20 h in a time dependent manner. On the other hand, the level of lamin-B1 protein in F28-7-A continued to increase until 16 h of the FUdR-treatment, then it decreased gradually, until it reached a low level at 12–20 h. Previous report has indicated that lamin-B1 undergoes caspase-mediated degradation during apoptosis [26]. These findings suggest that the differential expression pattern of lamin-B1 determines the FUdR-induced cell death mode. In addition, we reported previously that the lamin-B1 protein level is 1.8-fold higher in F28-7 than in F28-7-A cells [20, 22]. However, the levels of lamin-B1 mRNA expressions were equal in these sister cells [21]. This suggests that the lamin-B1 regulation by miR-351 may reside in the translation stage. Of note, lamin-B1 is not a validated target of miR-351 (miRBase: http://www.mirbase.org/). It is known (TargetScan: http://www.targetscan.org/) that the miR-351-5p has a seed sequence with "UCCCUGAG" near its 5'-end. This sequence is essential for this mimic to bind to its target mRNA [27]. Bioinformatic analysis shows that the miR-351-5p binding sequence “CUCAGGG” is not present in 3’-UTR of lamin-B1 mRNA sequence. We became aware that this miR351-5p's possible binding region is present in lamin-B1 mRNA (1904–1911, CUCAGGGA) (Fig 6). Therefore, we hypothesize that the protein expression of lamin-B1 may be suppressed by miR-351-5p in the translational stage. In addition, we think it possible that the observed lamin-B1 depression in the miR-351 transfected cells was indirectly mediated by miR-351 targeting of other factors (*e*.*g*., translation related proteins). We are currently investigating possible interactions between miRNA-351 and lamin-B1 mRNA by cell-free *in vitro* pull-down assay using synthetic miRNA-351-5p and biotinylated lamin-B1 partial mRNA (details to be published elsewhere).

It would be important to further investigate possible management of lamin-B1 expression by miR-351. Here, we showed that the “overexpression” of miR-351 in necrosis-fated cells leads to reduction of lamin-B1 expression, accompanying a shift from necrotic morphology to apoptotic morphology. We also plan to investigate miR-351 functions in these two types of cell death processes by use of F28-7 and F28-7-A cells having equal miR-351 expression levels.

We previously reported that a decrease in lamin-B1 intermediate filament-protein gives rise to greater flexibility in nucleus and cell structure, thereby leading to apoptosis [22]. It is noteworthy that Tang *et al*. reported that a reduced expression of lamin-B1 by siRNA severely inhibits RNA synthesis [28]. In addition, Butin-Israeli *et al*. recently suggested that the maintenance of lamin-B1 levels is required for DNA replication and repair through regulation of the expression of key factors, including BRCA1 and RAD51, involved in these essential nuclear functions [29]. We consider that a decrease in lamin-B1 expression may alter not only membrane flexibility but also compositions of intracellular environment that determines whether the type of FUdR-induced cell death is necrosis or apoptosis.

In recent years, several miRNAs were used for cancer diagnosis and therapy [30, 31]. The miRNA-351, a species-specific microRNA, is found only in mouse and rat. The miR-351 belongs to the miR-125 family, and is shown to perform various roles in cell development, cell death, cancer and inflammation [32–35]. Shomron and coworkers suggest that the miR-125 family members possess identical seed sequences, which are predicted to share the same target genes [27]. It is known that miRNA-351 and miR-125a, members of a human miRNA family, play a critical role in mitochondrial autophagy (mitophagy) and erythropoiesis [36–38]. Our previous work revealed that release of cytochrome c from mitochondria into cytoplasm and nucleus occurs in the apoptosis but not in the necrosis [22]. Interestingly, miR-125 plays important roles in mitochondrial apoptosis pathway by targeting pro-apoptosis or anti-apoptosis genes depending on cellular environment [39]. It was reported that a treatment with FUdR reduced mitochondrial DNA in HeLa and CEM T-lymphoblastic cell lines [40, 41]. We consider that miR-351 regulates not only the expression of lamin-B1 but also the expression of other cell-death regulators in the necrosis-apoptosis. It is possible that the two-types of cancer cell-death, necrosis and apoptosis, are regulated by miR-125 family members in various living species. We further demonstrate an association of miR-351 and mitochondrial events (including mitophagy and mitochondrial DNA synthesis) in FUdR-induced cell death.

### Conclusion

We thus identified a new regulator of the cell death, the microRNA miR-351. This finding would be important in exploring roles of miRNA(s) in the cell-death processes.

## Supporting Information

S1 Fig Higher expression of miR-743a shifts the FUdR-induced cell death morphologies from necrosis to apoptosis. (A) F28-7 cells were transfected with nonsilencing siRNA (NSsi), and with mature miR-743a-3p mimic (miR743am). At 48 h after the transfection, the levels of miR-743a-3p and RNU6B (an internal standard) were analyzed by quantitative real-time PCR. Results are averages of three independent experiments with error bars showing the ±SD in triplicates. The asterisk indicates a statistically significant difference (Student’s *t* test, *p*0.05). (B) At 48 h after transfection with the mature miR-743a-3p mimic (miR743am), the F28-7 cells were treated with or without 1 μM FUdR for 21 h, and then stained with DAPI. Morphological changes were analyzed by Olympus BX61 fluorescence microscope at 400× magnification.
(PDF)

S2 Fig Dynamics of lamin-B1 protein in FUdR-induced necrosis and apoptosis. Whole cell lysates were prepared from F28-7 and F28-7-A cells in untreated (0h) and FUdR-treted stages (4, 8, 12, 16, and 20 h). Expression of lamin-B1 and GAPDH proteins were analyzed by western blotting. Expression of GAPDH was used as an internal control. The results shown are those of an individual experiment that was representative of three independent experiments.
(PDF)
