# Supplementary figures and images for: MicroRNA-351 Regulates Two-Types of Cell Death, Necrosis and Apoptosis, Induced by 5-fluoro-2'-deoxyuridine
Source: PLoS One. 2016 Apr 12;11(4):e0153130. doi: 10.1371/journal.pone.0153130 (PMC4829180; doi:10.1371/journal.pone.0153130)

**A**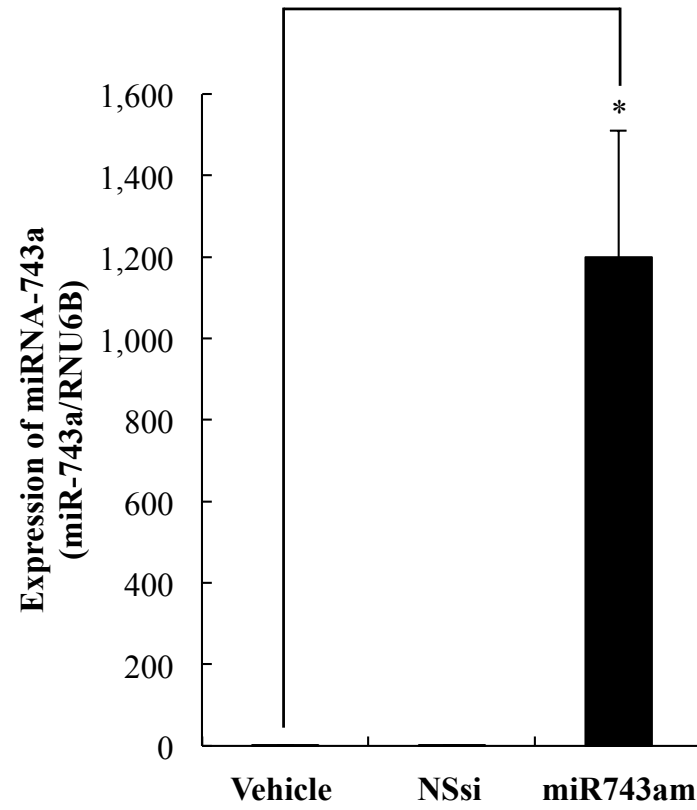**B**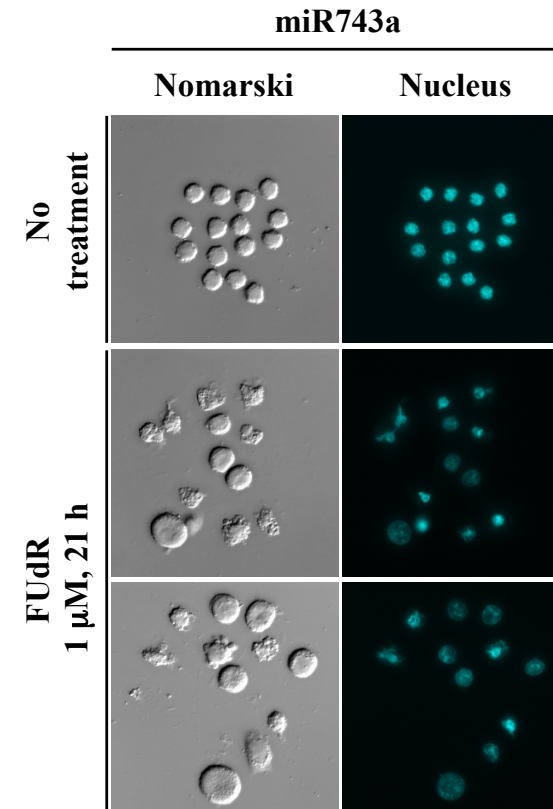

Supplement: S1 Fig — (A) F28-7 cells were transfected with nonsilencing siRNA (NSsi), and with mature miR-743a-3p mimic (miR743am). At 48 h after the transfection, the levels of miR-743a-3p and RNU6B (an internal standard) were analyzed by quantitative real-time PCR. Results are averages of three independent experiments with error bars showing the ±SD in triplicates. The asterisk indicates a statistically significant difference (Student’s t test, p0.05). (B) At 48 h after transfection with the mature miR-743a-3p mimic (miR743am), the F28-7 cells were treated with or without 1 μM FUdR for 21 h, and then stained with DAPI. Morphological changes were analyzed by Olympus BX61 fluorescence microscope at 400× magnification. (PDF) [file pone.0153130.s001.pdf]

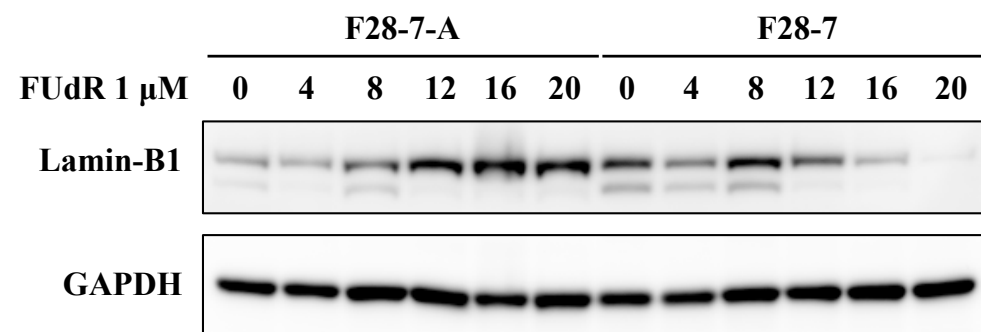

Supplement: S2 Fig — Whole cell lysates were prepared from F28-7 and F28-7-A cells in untreated (0h) and FUdR-treted stages (4, 8, 12, 16, and 20 h). Expression of lamin-B1 and GAPDH proteins were analyzed by western blotting. Expression of GAPDH was used as an internal control. The results shown are those of an individual experiment that was representative of three independent experiments. (PDF) [file pone.0153130.s002.pdf]
